# Cold Air for Domestic Uses

**Published:** 1881-06

**Authors:** 


### COLD AIR FOR DOMESTIC USE.

The *Chronique Industrielle* gives an abstract of a paper by a French engineer, M. Mougey, of Bray-sur-Seine, wherein the author shows the benefits to be derived from a system proposed by him for distributing cold air through a line of pipes to private consumers. Some such system has been suggested before, but the one under consideration differs from it in the fact that the projector proposes to compress the air to a greater degree (5 or 6 atmospheres), and to *cool* it before sending it through the pipes to the various points of distribution. At these points the opening of a cock, by allowing the air to escape and expand, will distribute throughout cellars, living apartments, or wherever else it may be needed, a pure cold air capable of preventing fermentation or putrefaction of organic matters, and of rendering the atmosphere of stores, manufactories, or dwelling houses refreshing during the most sultry days of summer. The air thus compressed may also be used, like steam, as a motive power. As for the proposed mode of distribution, that is essentially the same as now employed for supplying steam heat to consumers in Lockport, N. Y.—*Scientific American.*

